# Epigenetic Regulation of Matrix Metalloproteinase-1 and -3 Expression in *Mycobacterium tuberculosis* Infection

**DOI:** 10.3389/fimmu.2017.00602

**Published:** 2017-05-24

**Authors:** Rachel C. Moores, Sara Brilha, Frans Schutgens, Paul T. Elkington, Jon S. Friedland

**Affiliations:** ^1^Section of Infectious Diseases and Immunity, Imperial College London, London, UK; ^2^Centre for Inflammation and Tissue Repair, Respiratory Medicine, University College London, London, UK; ^3^National Institute of Health Research (NIHR) Respiratory Biomedical Research Unit, Faculty of Medicine, University of Southampton, Southampton, UK

**Keywords:** *Mycobacterium tuberculosis*, matrix metalloproteinases, histone deacetylases, histone acetyltransferases, epigenetics

## Abstract

In pulmonary tuberculosis (TB), the inflammatory immune response against *Mycobacterium tuberculosis* (Mtb) is associated with tissue destruction and cavitation, which drives disease transmission, chronic lung disease, and mortality. Matrix metalloproteinase (MMP)-1 is a host enzyme critical for the development of cavitation. MMP expression has been shown to be epigenetically regulated in other inflammatory diseases, but the importance of such mechanisms in Mtb-associated induction of MMP-1 is unknown. We investigated the role of changes in histone acetylation in Mtb-induced MMP expression using inhibitors of histone deacetylases (HDACs) and histone acetyltransferases (HAT), HDAC siRNA, promoter-reporter constructs, and chromatin immunoprecipitation assays. Mtb infection decreased Class I HDAC gene expression by over 50% in primary human monocyte-derived macrophages but not in normal human bronchial epithelial cells (NHBEs). Non-selective inhibition of HDAC activity decreased MMP-1/-3 expression by Mtb-stimulated macrophages and NHBEs, while class I HDAC inhibition increased MMP-1 secretion by Mtb-stimulated NHBEs. MMP-3 expression, but not MMP-1, was downregulated by siRNA silencing of HDAC1. Inhibition of HAT activity also significantly decreased MMP-1/-3 secretion by Mtb-infected macrophages. The MMP-1 promoter region between −2,001 and −2,942 base pairs from the transcriptional start site was key in control of Mtb-driven MMP-1 gene expression. Histone H3 and H4 acetylation and RNA Pol II binding in the MMP-1 promoter region were increased in stimulated NHBEs. In summary, epigenetic modification of histone acetylation via HDAC and HAT activity has a key regulatory role in Mtb-dependent gene expression and secretion of MMP-1 and -3, enzymes which drive human immunopathology. Manipulation of epigenetic regulatory mechanisms may have potential as a host-directed therapy to improve outcomes in the era of rising TB drug resistance.

## Introduction

Tuberculosis (TB) remains a major global health problem, with 10.4 million new cases and 1.8 million deaths per year (1). The rapid emergence of widespread drug resistance necessitates new strategies to improve the efficacy of treatment in TB, both to decrease transmission and to improve patient outcomes. Ideally, such therapies will shorten the duration of therapy, which is currently a minimum of 6 months and may be years in drug-resistant disease. Host-directed therapies are increasingly of interest in TB (2).

The primary site of *Mycobacterium tuberculosis* (Mtb) infection is the lung, and pulmonary disease is characterized by granulomatous inflammation with destruction of lung parenchyma. The outcome of infection is very variable between hosts, and the factors determining this are not well understood, although host genetics and innate immune responses are important determinants of disease (3, 4). Matrix metalloproteinases (MMPs) are zinc-dependent endopeptidases, which have key roles in tissue repair and in diseases characterized by inflammatory tissue destruction such as emphysema (5). MMPs are key mediators of inflammatory cell migration, and modulators of chemokine and cytokine signaling (6, 7). MMP activity is strongly implicated in the immunopathogenesis of TB. Our group and others demonstrated the involvement of MMP-1, the major human collagenase, and its activator MMP-3 (stromelysin-1) in driving pathology in pulmonary TB (8–10). MMPs are secreted by Mtb-infected monocytes and macrophages, and also by uninfected stromal cells stimulated via intercellular networks (11). Epigenetic mechanisms are emerging as major regulators of MMP activity in non-infectious diseases (12, 13), including chronic lung diseases such as asthma and COPD (14), but their role in MMP expression in TB is less established.

Epigenetic regulation encompasses all chromosomal modifications that alter gene expression without altering the nucleotide sequence of coding DNA (15, 16). Eukaryotic DNA is packaged as chromatin around octamers of histone proteins, which contain globular domains and negatively charged tails. These are subject to extensive post-translational modification, including acetylation of highly conserved lysine residues. Acetylation of histones H3 and H4, carried out by Histone acetyltransferases (HATs), is associated with increased gene transcription (17). Conversely, acetyl groups are removed by the histone deacetylases (HDACs), which are divided into four classes. The Class I HDACs (1, 2, 3, and 8) are ubiquitously expressed, whereas Class II HDACs (such as HDAC 4 and 5) are selectively expressed in different tissues. HDAC activity is usually associated with silencing of gene expression (18, 19). However, this is not uniformly the case and opposite regulation may occur. For example, HDAC inhibition reduced MMP-9 gene expression in cancer cell lines resulting in a less invasive phenotype (20).

The epigenetic mechanisms regulating inflammatory immune responses in TB are an emerging field. Altered miRNA expression in serum and sputum from TB patients compared to controls has been shown, and potential biomarkers for diagnosis have been identified (21). A growing body of evidence exists to support the importance of epigenetic mechanisms in other respiratory infections, for example, altered DNA methylation patterns in asthma patients versus healthy controls have been implicated in the pathogenesis of rhinovirus infection (22). Similarly, altered DNA (cytosine-5-)-methyltransferase-1 (DMT-1) expression in nasal epithelial cells from smokers was identified as a possible mechanism of increased susceptibility to influenza (23). *In vitro* studies of airway epithelial cells demonstrated increased HDAC2 expression and decreased histone acetylation in respiratory syncytial virus (RSV)-infected cells, while chemical HDAC inhibition restricted RSV replication (24). In the current study, we have investigated whether epigenetic modifications, specifically histone acetylation/deacetylation, regulated the characteristic TB-associated expression of MMP-1 and MMP-3 by monocyte-derived macrophages and normal human bronchial epithelial cells (NHBEs), thereby augmenting TB immunopathology. The role of histone acetylation in induction of MMP-1/-3 expression was specifically investigated, since this dynamic epigenetic mark is associated with transcriptional activation. We demonstrate that Mtb infection alters macrophage Class I HDAC expression and that MMP-1 expression induced by Mtb is sensitive to HDAC/HAT inhibition. In addition, increased histone acetylation was seen at MMP-1 and -3 promoter regions compared with unstimulated cells, specifically in the region −2,001 to −2,942 bp of the MMP-1 promoter, which contains key inducible sites activated in Mtb-stimulated cells.

## Materials and Methods

### Reagents and Antibodies

Trichostatin A (TSA) was purchased from Sigma-Aldrich (Gillingham, UK), CBHA, HAT inhibitor II and Anacardic acid (AA) from Calbiochem (Millipore, Watford, UK), and MS-275 from Enzo Life Sciences (Exeter, UK). Primary rabbit anti-human acetyl-histone H3 and acetyl-histone H4 (Millipore) were used for chromatin immunoprecipitation. Primary mouse anti-human HDAC4 and anti-HDAC7 were used for Western blot and HRP-linked goat anti-rabbit IgG and goat anti-mouse (all from Cell Signalling, Hertfordshire, UK) were used as secondary antibodies. All other reagents were purchased from Sigma-Aldrich unless otherwise stated.

### Mtb Culture

*Mycobacterium tuberculosis* strain H37Rv was cultured from frozen stocks stored at −80°C in Middlebrook 7H9 broth (BD Biosciences, Oxford, UK) supplemented with 10% OADC enrichment medium (BD Biosciences), 0.2% glycerol, and 0.02% Tween 20 with agitation at 37°C. Growth was monitored by measuring optical density (OD) using a Biowave cell density meter (WPA, Cambridge, UK). Infection experiments were performed using cultures at mid-log growth (at OD 0.55–0.65) corresponding to 1–2 × 10^8^ CFU/ml. Correlation with optical density was checked by performing colony counts in triplicate on Middlebrook 7H11 agar. Cells were infected at a multiplicity of infection (MOI) of 1 unless otherwise stated.

### Cell Culture

Monocytes were adhesion-purified from leukocyte residues from healthy blood donors (NHS Blood Transfusion Service) and differentiated into macrophages for 4 days in RPMI 1640 (Life Technologies, Paisley, UK) supplemented with 10% fetal bovine serum (FBS) and 100 ng/ml M-CSF (R&D Systems, Abingdon, UK). After a further 24 h without M-CSF, the medium was changed for M-SFM (Life Technologies) and cells were infected with Mtb H37Rv strain. Cells were pretreated with chemical inhibitors for 2 h prior to infection where relevant.

Primary NHBEs (Lonza, Wokingham, UK) were cultured according to the supplier’s instructions in supplemented bronchial epithelial growth medium (BEGM). Medium was replaced every 3 days. Cells were subcultured at 80% confluence and used at passage 4 or 5.

The alveolar carcinoma cell line A549 (ATCC, Middlesex, UK) was cultured in RPMI 1640 supplemented with 2 mM glutamine, 10 µg/ml ampicillin, and 10% FBS. For experiments, cells were seeded at 4 × 10^4^ cells/cm^2^ and stimulated 24 h later.

### Conditioned Medium from *Mycobacterium tuberculosis*-Infected Monocytes (CoMTb)

Peripheral blood monocytes isolated as above from healthy blood donors were infected with H37Rv at a MOI of 1 in RPMI without FBS for 24 h. The culture medium was then collected and sterilized by passage through a 0.2 µM Anopore syringe filter (Whatman, Brentford, UK). Paired samples of conditioned medium from uninfected monocytes (CoMCont) from the same donor were used as controls.

### MMP ELISAs

Supernatants were collected at 72 h poststimulus, sterile filtered, and MMPs were quantified using the Duoset MMP-1 and MMP-3 ELISA kits (R&D Systems) according to manufacturer’s instructions. Lower limits of sensitivity for the Duoset kits are: 156 pg/ml for MMP-1 and 31.2 pg/ml for MMP-3. Samples were run with appropriate controls and at dilutions calculated to give readings within the linear range of detection as indicated by the manufacturer.

### Luminex Multiplex Immunoassay

Quantification of MMP-1, -3, -7, and -9 concentrations was performed using the Fluorokine MultiAnalyte Profiling MMP Base Kit (R&D Systems) and the Luminex platform Bio-Plex 200 (Bio-Rad, Hemel Hempstead, UK) dual laser analyzer. Standard curves were generated using Bio-Plex Manager version 5.0. Lower limits of sensitivity for the Fluorokine Luminex were: 1.1 pg/ml for MMP-1, 7.3 pg/ml for MMP-3, 6.6 pg/ml for MMP-7, and 13.7 pg/ml for MMP-9. All samples were run with appropriate controls and were within the linear range of detection as indicated by the manufacturer.

### Western Blotting

Cells were washed in PBS and scraped and homogenized in lysis buffer (62.5 mM Tris, 2% SDS, 10% glycerol, 50 mM DDT with bromophenol blue) prior to storage at −80°C. Samples were denatured by heating to 90°C for 5 min and loaded on 4–12% NuPAGE Bis-Tris mini gels (Life Technologies) and run at 200 V for 70–90 min. Proteins were transferred to a nitrocellulose membrane (GE Healthcare) at 4°C, 30 V, for 1 h before blocking with 5% skimmed milk protein/TBS/0.1% Tween 20 buffer and incubating with diluted primary antibody. Membranes were incubated with diluted HRP-linked secondary antibody and developed using ECL Prime developing kit (GE Healthcare, Hatfield, UK).

### Transient Transfection with Promoter-Reporter Constructs

MMP-1 promoter constructs expressed in the pGL3 firefly (*Photinus pyralis*) luciferase expression vector (Promega, Southampton, UK) were a gift of Professor Ian Clark (University of East Anglia, Norwich, UK). The full-length wild-type (WT) MMP-1 promoter construct (WT) comprised a 4,372 bp sequence upstream of the MMP-1 transcriptional start site. Deletion constructs ranged in size from 3,830 to 517 bp (25). MMP-3 promoter constructs were designed in-house and cloned into the pGL3 vector. Truncations were generated using primers that incorporated restriction enzyme sites within the sequence of interest: MMP3-1R 5′-GCTTTACTTAGATCTATGTTGTCTC-3′; MMP3-4F 5′-GCTAGAGCTAGCAAGGATCCTGCAC-3′; MMP3-6R 5′-CTTCATTTCCACAAGCTTTACTTAGCTCT-3′; MMP3-7F 5′-GTTTTCCTCCTCGAGAACCAGCAAATCC-3′; MMP3-8F 5′-CATCATTCTACTGAGCTCTTACTCCCAAG-3′; MMP3-9F 5′-CCATGTCTGTAATCCTAGCACTTTGAG-3′; MMP3-10F 5′-GTTCAGTGTGGAAAATAGAGTAGCAGAGG-3′; MMP3-11—F 5′-GATGGATTCTCGAGTTCAACTTCAAAGCATCTG-3′; MMP3-12—R 5′-GAGACAGAGATCTCACTATGTTGCCC-3′. PCR products of the 3 kb MMP-3 promoter region were digested in one step using *Nhe*I and *Bgl*II, followed by *Bam*HI and *Bgl*II for cloning into pGL4 and pBSK vectors. Shorter fragments of the original 3kb MMP-3 promoter region were digested using *Hin*dIII and *Xho*I for MMP3-7F/MMP3-6R; *Sac*I and *Hin*dIII for MMP3-8F/MMP3-6R and MMP3-9F/MMP3-6R, and *Kpn*I and *Hin*dIII for MMP3-10F/MMP3-6R. After 2 h incubation at 37°C, enzymes were inactivated at 68°C for 20 min. The constructs generated were 2,183, 1,612, and 642 bp in length. WT MMP-1/-3 promoters and respective truncations were inserted upstream of the luciferase reporter gene in the pGL3 vector. The PRL-TK plasmid constitutively expressing *Renilla* luciferase was used to control for transfection efficiency.

A549 cells were transfected when 60% confluent with FuGene 6 (Roche, Lewes, UK), and 0.8 µg plasmid DNA or control plasmid DNA. Sixteen hours after transfection, the cells were stimulated according to the experimental conditions. Twenty-four hours later, cells were washed once in PBS and lysed in passive lysis buffer (Promega). Luciferase assays were performed using the Promega Dual-Luciferase Reporter Assay kit (Promega) using an L-Max 2 luminometer (Molecular Devices, Sunnydale, CA, USA).

### Real-time PCR

After 24 h incubation in the specified experimental settings, cells were lysed in TRI-reagent and total RNA extracted with the PureLink RNA mini kit (Life Technologies) with on-column DNase treatment. RNA concentrations and purity were evaluated using a Nanodrop spectrophotometer (Thermo Scientific, Wilmington, DE, USA). A total of 1 µg of sample RNA was reverse transcribed using the Quantitect RT Kit (Qiagen, Crawley, UK) according to the manufacturer’s instructions. Real-time PCR was performed using Brilliant II qPCR mastermix (Agilent, UK) on a Stratagene Mx3000p platform (Stratagene, La Jolla, USA). The thermal profile was 10 min at 95°C, followed by 40–45 cycles of 30 s at 95°C and 1 min at 60°C. The cycle threshold (Ct) at which amplification entered the exponential phase was determined for each well and analyte. 18S ribosomal RNA, β-actin, and GAPDH RNA were used as reference genes. The following primers and probes were used to analyze target and reference genes.

MMP-1 forward primer 5′-AAGATGAAAGGTGGACCAACAATT-3′; reverse primer 5′-CCAAGAGAATGGCCGAGTTC-3′ and probe 5′-FAM CAGAGAGTACAACTTACATC GTGTTGCGGCTC-TAMRA-3′; GAPDH forward primer 5′-CGCTTCGCTCTCTGCTCC T-3′; reverse primer 5′-CGACCAAATCCGTTGACTCC-3′ and probe 5′-HEX-CGTCGCC AGCCGAGCCACAT-TAMRA-3′ (both from Sigma-Aldrich); MMP-3 Hs00968305_m1; HDAC1 Hs02621185_s1; HDAC2 Hs00231032_m1; HDAC3 Hs0018730_m1; HDAC4 Hs01041638_m1; HDAC5 Hs00608366_m1; HDAC8 Hs00218503_m1; 18S 4308329 and β-actin 431088E (all Taqman primer and probe mixes from Life Technologies). Cts from target genes were normalized to Cts for the reference genes that were measured simultaneously for each PCR assay performed.

### Transfection of Epithelial Cells with siRNA

ON-TARGETplus SMARTpool siRNA oligonucleotides and transfection reagents were purchased from Dharmacon (Thermo Scientific). NHBEs were cultured in complete medium and transfected at 60–70% confluence. siRNA and Lipofectamine 2000 were diluted in Optimem and used at final concentrations of 30 nM and 25 µg/ml, respectively. After 4 h, each well was washed with PBS and fresh BEGM was added. The cells were then rested overnight prior to stimulation with the experimental conditions. For analysis of mRNA expression, cells were lysed and total RNA was extracted 24 h after stimulation. For analysis of protein expression, cell culture supernatants were collected for ELISA and cells were washed in PBS and lysed in Western lysis buffer 48 h poststimulation.

### Chromatin Immunoprecipitation Assay (ChIP)

Magna-ChIP kits and antibodies were purchased from Millipore (Watford, UK) and used according to manufacturer’s instructions. The rabbit polyclonal anti-acetyl-Histone H4 antibody was raised against tetra acetylated H4 and recognizes intermediately acetylated H4, but not acetylation on lysine 16. The rabbit polyclonal anti-acetyl-Histone 3 antibody was raised against acetylated N-terminus of H3.

Cells were cultured in 100 or 150 mm tissue culture dishes until confluent then stimulated as previously described. Cells were fixed with fresh 1% paraformaldehyde solution for 10 min and the reaction was stopped with glycine. Chromatin was sheared by sonication with a Covaris S2 ultra-sonicator using an Adaptive Focused Acoustics intensifier (KBioscience). Settings were as follows: temperature 6–8°C, duty cycle 20%, intensity 8, 200 cycles/burst, 15 cycles of 30 s, 2 × 10^7^ cell equivalents/ml. Immunoprecipitation was performed overnight at 4°C with 1 × 10^6^ cell equivalents per condition. Purified DNA was eluted and PCR performed using SYBR Green JumpStart Taq Readymix. Reactions were performed in triplicate on the Stratagene Mx3000P platform (Stratagene, La Jolla, USA). The following custom unlabeled primers were designed in-house and supplied by Sigma. MMP-1 transcription start site forward primer 5′-TGGGATATTGGAGCAGCAAG-3′and reverse primer 5′-AGCTGTGCATACTGGCCTTT-3′ (product size 82 bp); -500 bp MMP-1 promoter forward primer 5′-TAAGGGAAGCCAT GGTGCTA-3′; reverse primer 5′-AGGTTCCCTTCTGCCTTTGT-3′ (product size 65 bp); −2 kbp MMP-1 promoter forward primer 5′TTGCCAGATGGGACAGTGTA-3′ and reverse 5′-TCAGGAAAGCAGCATGTGAC-3′ (product size 123 bp); −4 kbp MMP-1 promoter forward primer 5′-CTTGAGGCCAGGAGTTTGAG-3′ and reverse primer 5′-ACCACCAT GTCCCACTGATT-3′ (product size 89 bp).

These assays were not performed in technical triplicates due to the number of cells required per condition.

### Statistics

Unless otherwise stated, results shown are from experiments performed in triplicate and representative of at least two independent experiments. Comparisons between two groups were made using the Student’s *t*-test (two-tailed with significance set as *p* < 0.05). For comparison of three or more groups, one-way ANOVA was used with Tukey’s correction for multiple comparisons. Unless otherwise stated in figure legends, graphs show mean values for triplicate samples and error bars are the SD.

## Results

### Class I HDACs Are Suppressed by Mtb Infection of Macrophages

**Figure 1:**
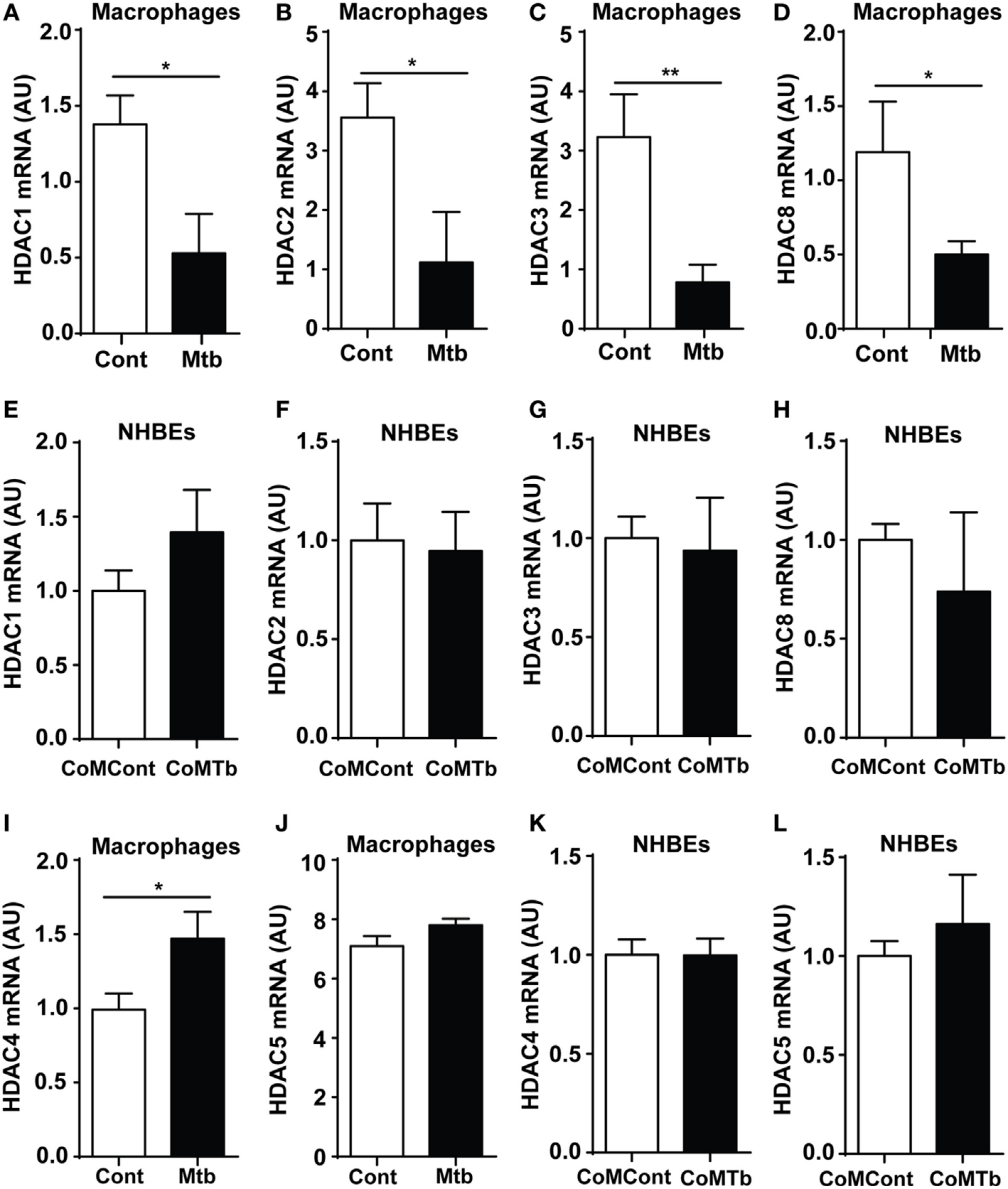
**Class I histone deacetylase (HDAC) expression is downregulated in *Mycobacterium tuberculosis* (Mtb)-infected macrophages**. Macrophages were infected with H37Rv at a multiplicity of infection of 1, while normal human bronchial epithelial cells (NHBEs) were stimulated with CoMTb (1:5 dilution), for 24 h before total RNA was extracted for Class I HDAC gene expression analysis. Figures show mRNA levels (AU) for: **(A,E)** HDAC1, **(B,F)** HDAC2, **(C,G)** HDAC3, **(D,H)** HDAC8 for macrophages and NHBEs, respectively. For Class II HDAC analysis, RNA was processed in a similar manner and figures show an **(I,K)** HDAC4 mRNA and **(J,L)** HDAC5 mRNA accumulation for macrophages and NHBEs. Bars represent mean ± SD and analysis was performed using Student’s *t*-test. *p < 0.05; **p < 0.01. AU, arbitrary units.

Experiments were designed to investigate epigenetic regulation of TB immunopathology, first investigating whether Mtb infection of macrophages altered Class I HDAC expression. Expression of Class I HDACs was repressed by Mtb infection, with a 68% decrease in HDAC1, 69% in HDAC2, 76% in HDAC3, and 58% in HDAC8 compared with uninfected controls (all *p* < 0.05) (Figures 1A–D). Next, we examined monocyte network-dependent stimulation of NHBEs by CoMTb. In contrast to macrophage stimulation by Mtb, CoMTb stimulation of NHBEs did not significantly alter accumulation of any class I HDAC mRNAs (Figures 1E–H). Expression of Class II HDACs 4 and 5 was also examined by RT-PCR. HDAC4 mRNA accumulation was increased by 48% in Mtb-infected macrophages, while no difference was observed for HDAC5 (Figures 1I,J). Increase in HDAC4 protein level was confirmed by western blot (Figure S1 in Supplementary Material). Class II HDAC expression in CoMTb-stimulated NHBEs was similar to control conditions (Figures 1K,L). Thus, exposure to live Mtb selectively suppressed macrophage class I HDAC expression.

### Non-Selective HDAC Inhibition Reduces MMP-1 and -3 Expression in Macrophages and NHBEs during Mtb Infection

**Figure 2:**
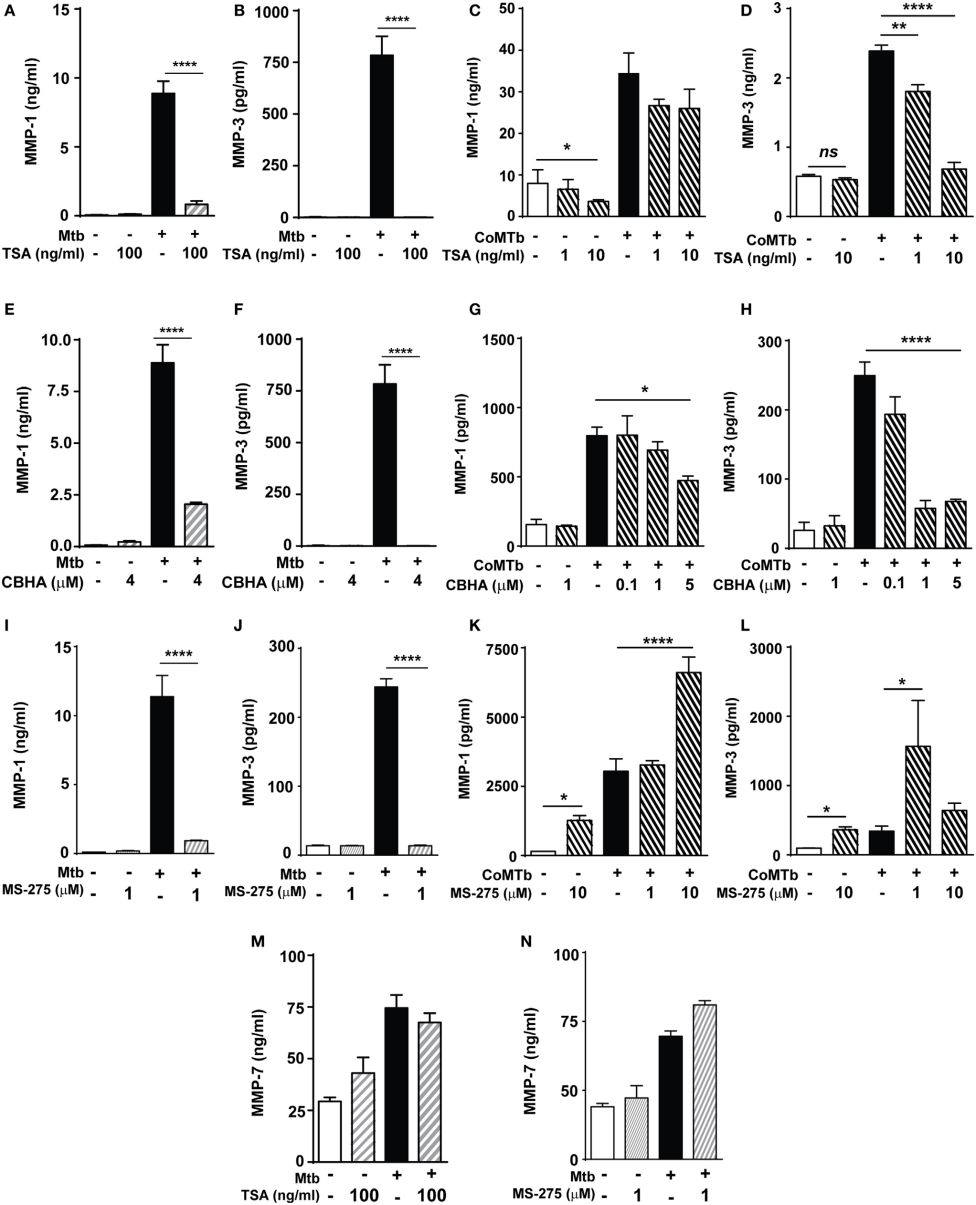
***Mycobacterium tuberculosis* (Mtb)-driven matrix metalloproteinase (MMP)-1 and -3 secretion is suppressed by histone deacetylase (HDAC) inhibition**. Macrophages and normal human bronchial epithelial cells (NHBEs) were preincubated with 1–100 ng/ml trichostatin A (TSA) **(A–D)**, 0.1–5 µM CBHA **(E–H)** or 1–10 µM MS-275 **(I–L)** to inhibit HDAC activity, prior to infection with H37Rv (multiplicity of infection 1) or stimulation with CoMTb (1:5 dilution) for 72 h, then MMP-1 and -3 secretion was measured. Pretreatment with TSA suppressed: **(A)** MMP-1 and **(B)** MMP-3 secretion from Mtb-infected macrophages. In CoMTb-stimulated NHBEs **(C)**, MMP-1 secretion was unaltered and **(D)** MMP-3 secretion decreased with TSA treatment. Pretreatment with CBHA suppressed: **(E)** MMP-1 and **(F)** MMP-3 secretion from Mtb-infected macrophages and **(G)** MMP-1 and **(H)** MMP-3 secretion from CoMTb-stimulated NHBEs. The Class I selective HDACi MS-275 suppressed: **(I)** MMP-1 and **(J)** MMP-3 secretion from Mtb-infected macrophages, while it increased **(K)** MMP-1 secretion and decreased **(L)** MMP-3 secretion from CoMTb-stimulated NHBEs. Secretion of MMP-7 was not affected by **(M)** TSA and **(N)** 1 µM MS-275 treatment in Mtb-infected macrophages. Bars represent mean ± SD and analysis was performed using one-way ANOVA with Tukey’s posttest. *p < 0.05; **p < 0.01; ****p < 0.0001. CBHA, m-Carboxycinnamic Acid bis-Hydroxamide.

Next, we investigated whether histone acetylation status affected MMP expression using the non-selective HDAC inhibitor (HDACi) TSA. A total of 100 ng/ml TSA markedly decreased Mtb-stimulated MMP-1 and MMP-3 secretion by macrophages (Figures 2A,B). In NHBEs, TSA significantly decreased baseline MMP-1 secretion by over 50% (*p* < 0.05; Figure 2C). CoMTb-stimulated MMP-1 secretion was decreased by TSA treatment, although this did not reach statistical significance (Figure 2C). In contrast, CoMTb-stimulated MMP-3 secretion was significantly reduced in a dose-dependent manner by 25% with 1 ng/ml TSA (from 2.4 to 1.8 ng/ml) and by 72% with 10 ng/ml TSA (685 pg/ml) (Figure 2D).

Further experiments were performed using an alternative chemical HDACi, CBHA (m-Carboxycinnamic Acid bis-Hydroxamide), to confirm that the observed effects were due to HDAC inhibition and not non-specific. Pretreatment of macrophages with 4 µM CBHA before Mtb infection decreased MMP-1 secretion by 77% (from 8.89 to 2.05 ng/ml) and MMP-3 secretion to undetectable levels (Figures 2E,F). CoMTb-driven MMP-1 secretion was reduced by CBHA treatment in a dose-dependent manner. 5 µM CBHA reduced MMP-1 secretion by 41% (from 797 to 473 pg/ml; *p* < 0.05; Figure 2G), while 1 µM CBHA was sufficient to completely inhibit CoMTb-induced MMP-3 secretion (*p* < 0.0001; Figure 2H). These results indicate that HDACs may differ between cell types, each with specific MMP regulatory pathways.

The HDAC class I-selective inhibitor MS-275 (1 µM) inhibited Mtb-driven macrophage MMP-1 secretion by 92% and reduced MMP-3 concentrations by 94.4% (Figures 2I,J). This supports the earlier finding and implies a key regulatory role for class I HDACs in macrophages MMP-1/-3 expression. In contrast, MS-275 enhanced MMP-1 secretion by both unstimulated (*p* < 0.05) and CoMTb-stimulated NHBEs (*p* < 0.0001; Figure 2K). MS-275 at a low concentration of 1 µM increased MMP-3 secretion 4.6-fold compared to CoMTb alone (*p* < 0.05), but this was not observed at the higher concentration of 10 µM MS-275 (Figure 2L), possibly due to sublethal cell toxicity, which could not be detected by cell viability experiments. The effects of these HDACi on MMP secretion were selective, since neither TSA nor 1 µM MS-275 significantly altered MMP-7 secretion by Mtb-infected macrophages (Figures 2M,N).

### Silencing HDAC1 Expression Does Not Affect CoMTb-Driven MMP-1 Expression

**Figure 3:**
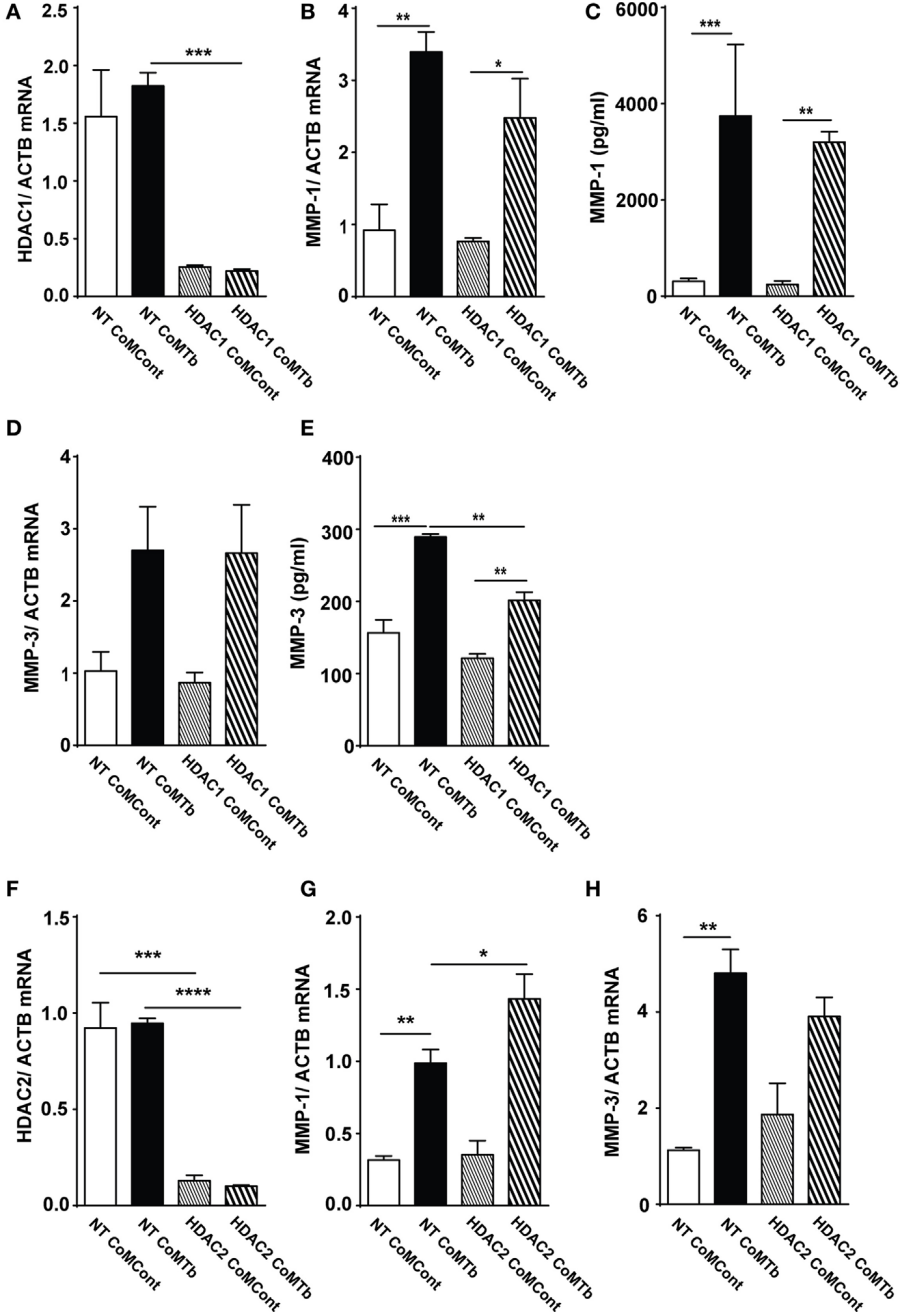
**Silencing of HDAC1 and HDAC2 expression does not inhibit CoMTb-driven matrix metalloproteinase (MMP)-1 gene expression**. Normal human bronchial epithelial cells (NHBEs) were transfected with 30 nM non-targeting (NT), HDAC1- or HDAC2-specific siRNA, or treated with transfection reagent alone. Cells were then stimulated with CoMTb (1:5) for 24 or 48 h. **(A)** HDAC1 mRNA normalized to the reference gene ACTB shows siRNA suppresses mRNA levels. **(B)** MMP-1 mRNA accumulation and **(C)** MMP-1 secretion following HDAC1 silencing are not significantly suppressed by siRNA. **(D)** MMP-3 mRNA accumulation remained unchanged, while **(E)** MMP-3 secretion decreased following HDAC1 silencing. **(F)** HDAC2 mRNA normalized to the reference gene ACTB and accumulation was silenced by HDAC2 siRNA. HDAC2 silencing increased MMP-1 mRNA accumulation **(G)** and did not affect MMP-3 mRNA accumulation **(H)** in CoMTb-stimulated NHBEs. mRNA of target genes was normalized to mRNA of the reference gene ACTB. Bars represent mean ± SD and analysis was performed using one-way ANOVA with Tukey’s posttest. *p < 0.05; **p < 0.01; ****p < 0.0001; ns, non-significant; ACTB, beta-actin; HDAC, histone deacetylase siRNA; NT, non-target siRNA.

Next, we investigated whether a specific class I HDAC enzyme was necessary for CoMTb-induced MMP-1 and -3 expression. HDAC1 mRNA was reduced by more than 80% in CoMTb-stimulated cells transfected with 30 nM HDAC1 siRNA compared to non-targeting (NT) siRNA or untransfected controls (Figure 3A; *p* = 0.001). Despite efficient HDAC1 silencing, no significant differences were observed in MMP-1 mRNA accumulation (Figure 3B). Consistent with this, MMP-1 concentrations were 3,204 pg/ml in CoMTb/HDAC1 siRNA treated samples compared to 3,746 pg/ml in the CoMTb/NT siRNA samples (Figure 3C). MMP-3 mRNA was upregulated threefold by CoMTb stimulation but no difference was observed between NT and HDAC1 siRNA-transfected NHBEs (Figure 3D). MMP-3 protein secretion in the NT-transfected cells was upregulated by CoMTb (*p* < 0.001), and MMP-3 secretion was reduced by 30% in HDAC1 siRNA-transfected cells compared to NT-transfected conditions (*p* < 0.01; Figure 3E). Silencing of HDAC2 in epithelial cells with siRNA (Figure 3F) was also effective and increased CoMTb-stimulated MMP-1, but not MMP-3, mRNA expression compared to CoMTb alone (*p* < 0.05; Figures 3G,H). Silencing HDAC3 with siRNA did not affect MMP-1 or MMP-3 expression (Figures S2A–C in Supplementary Material).

### Macrophage-Derived MMP-1 and -3 Gene Expression and Secretion during Mtb Infection Are Blocked by HAT Inhibition

**Figure 4:**
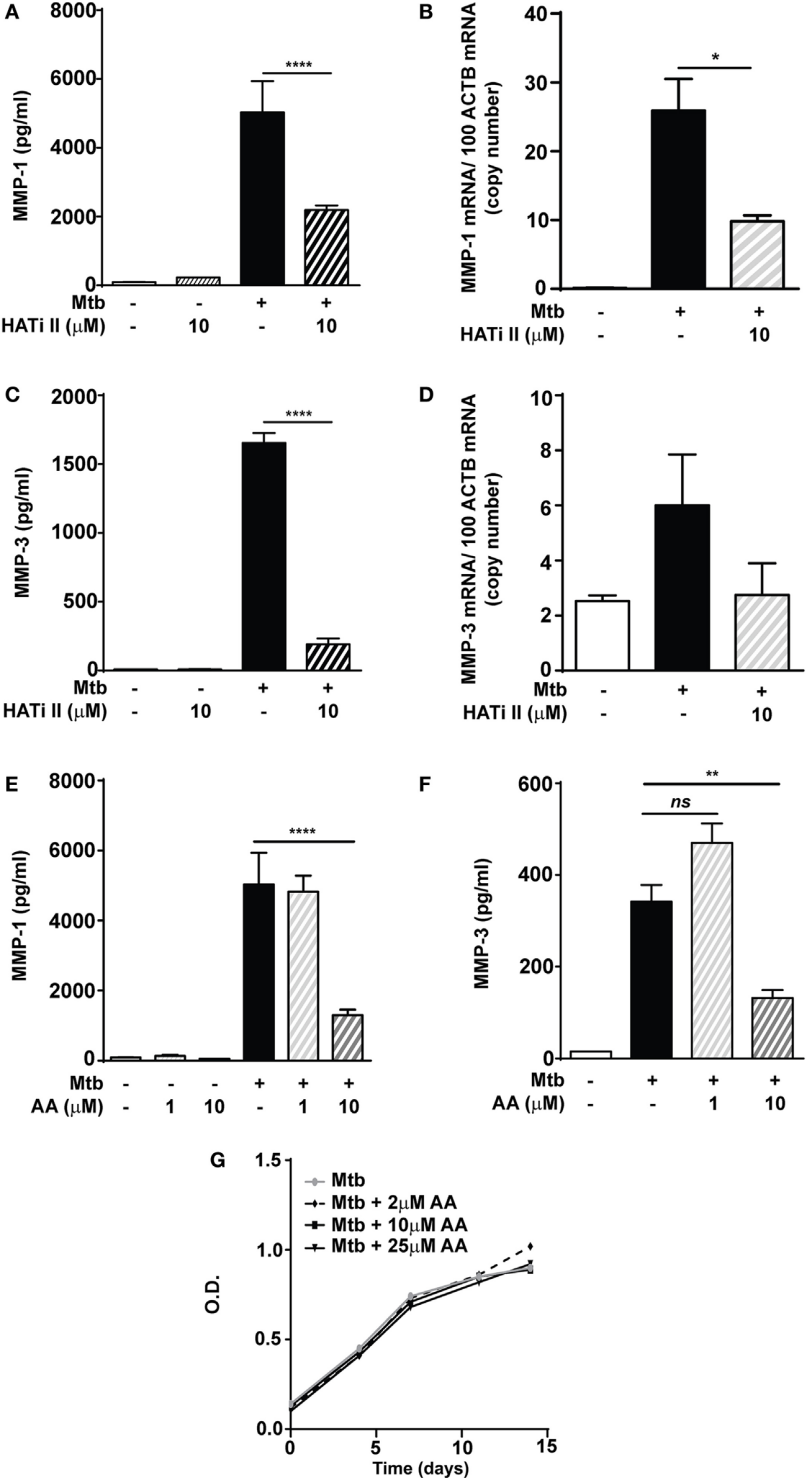
**HATi II inhibits *Mycobacterium tuberculosis* (Mtb)-driven matrix metalloproteinase (MMP)-1 and -3 secretion**. Macrophages were preincubated with 10 µM HATi II or 1–10 µM AA prior to infection with H37Rv (multiplicity of infection 1). Total RNA was extracted after 24 h and cell culture supernatants were collected after 72 h. Pre-treatment with the HATi II suppressed: **(A)** MMP-1 secretion and **(B)** MMP-1 mRNA accumulation, as well as **(C)** MMP-3 secretion and **(D)** MMP-3 mRNA accumulation. Pre-treatment with 1–10 µM AA suppressed: **(E)** MMP-1 and **(F)** MMP-3 secreted concentrations in Mtb-infected macrophages. **(G)** H37Rv was cultured in the presence of AA 2–25 µM and bacterial growth was accessed by optical density (OD) measurements. Bars represent mean ± SD and analysis was performed using one-way ANOVA with Tukey’s posttest. *p < 0.05; **p < 0.01; ****p < 0.0001; ns, non-significant; ACTB, beta-actin; AA, anacardic acid.

Next, the role of HAT activity was investigated using the inhibitor HATi II. 10 µM HATi II significantly decreased MMP-1 secretion from Mtb-infected macrophages by 56% (from 5,029 to 2,187 pg/ml) and mRNA accumulation by 62% (Figures 4A,B). MMP-3 secretion was decreased from 1,653 to 190 pg/ml (*p* < 0.0001, Figure 4C). There was a non-significant trend to decreased MMP-3 mRNA accumulation with HATi II treatment (Figure 4D). HATi II treatment did not affect MMP-7 secretion in response to Mtb infection (Figure S3 in Supplementary Material).

A second HAT inhibitor, AA (10 µM), reduced Mtb-infected macrophage MMP-1 secretion by 74% (from 5,029 to 1,302 pg/ml; *p* < 0.0001) (Figure 4E). Similarly, a significant decrease in MMP-1 secretion was also detected in stimulated NHBE cells pretreated with AA (Figure S4 in Supplementary Material). Secretion of MMP-3 in Mtb-infected macrophages was also inhibited by 10 µM AA (*p* < 0.01; Figure 4F). The AA compound is closely related to salicylic acid and has been reported to have some antimicrobial activity, including against Mtb (26, 27). We therefore investigated whether these results might be secondary to an effect on Mtb growth, but this was not altered in broth cultures containing AA at concentrations between 2 and 25 µM (Figure 4G). These experiments support the hypothesis that HAT activity is required for inducible expression of MMP-1 and MMP-3 in macrophages and NHBEs stimulated with Mtb.

### MMP-1 and MMP-3 Promoter-Reporter Analysis in TB

**Figure 5:**
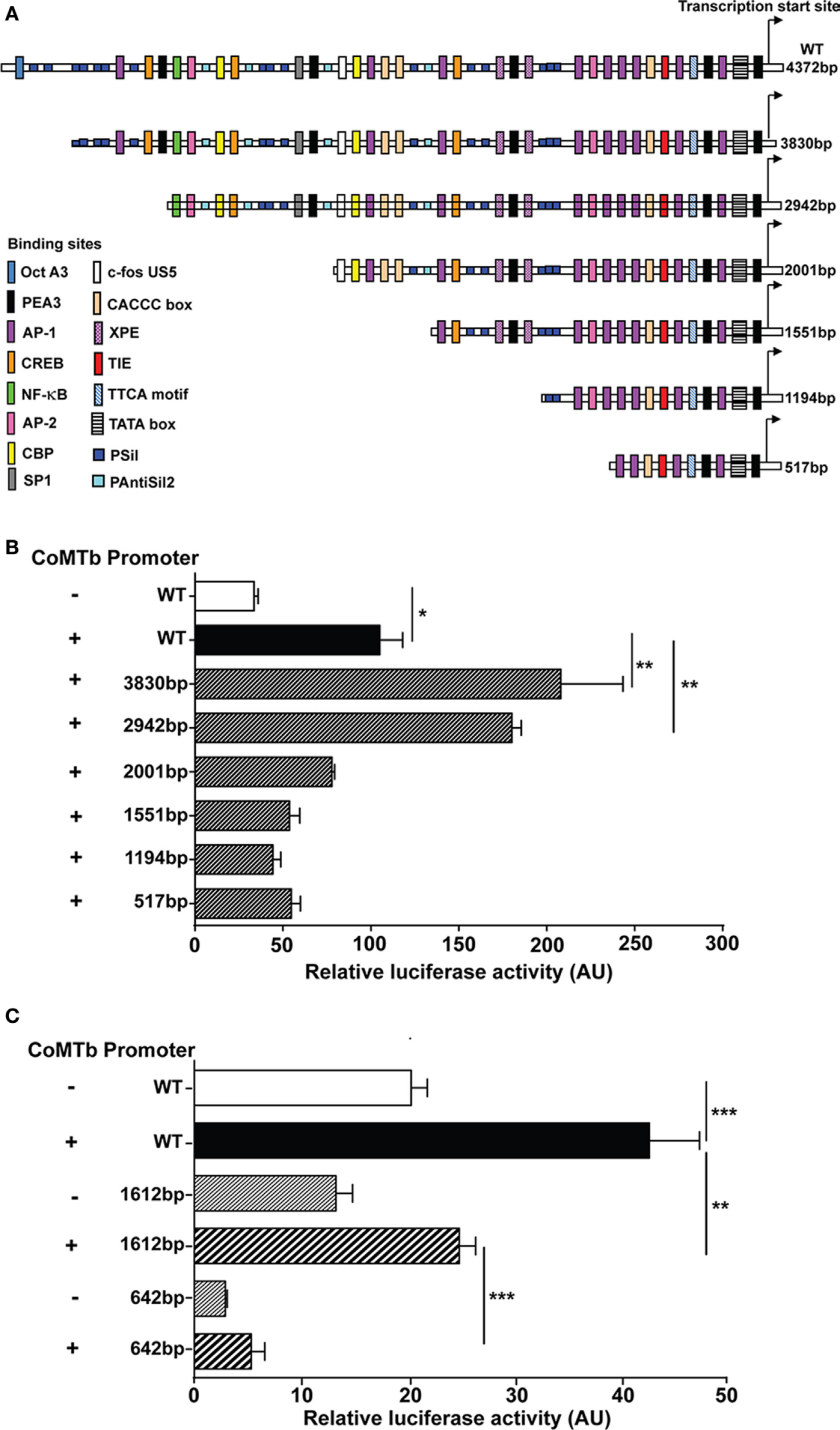
**Regulation of matrix metalloproteinase (MMP)-1 and -3 transcription in CoMTb-stimulated respiratory epithelial cells**. WT and truncations of the MMP-1 and MMP-3 promoters expressed in the pGL3 firefly luciferase expression vectors were transfected into A549 cells. MMP-1 and -3 promoter activity for each truncation was assessed by relative luciferase activity compared to WT controls. **(A)** Schematic representation of WT MMP-1 promoter and truncations and relevant transcription factors binding sites. **(B)** MMP-1 promoter activity is significantly repressed in truncations upstream −2,001 bp of the transcriptional start site. **(C)** MMP-3 promoter activity is suppressed by truncations upstream −1,612 bp from the transcriptional start site. Bars represent mean ± SD and analysis was performed using one-way ANOVA with Tukey’s posttest. *p < 0.05; **p < 0.01; ***p < 0.001. AU, arbitrary unit; WT, wild-type.

Plasmid promoter-reporter constructs of the MMP-1 and MMP-3 promoter regions were transfected into A549 respiratory epithelial cells to investigate the effect of Mtb-stimulation on promoter activity. A schematic representation of the relevant region of the human MMP-1 promoter region is shown in Figure 5A. CoMTb treatment increased promoter activity of the WT construct by more than threefold compared to controls (*p* = 0.02; Figure 5B). CoMTb-mediated promoter activation was significantly enhanced by 98 and 71% in the 3,830 and 2,942 bp constructs, respectively, compared to CoMTb-stimulated WT (*p* < 0.01). Further truncation of the promoter resulted in loss of CoMTb-driven promoter activity. The MMP-3 promoter, examined using similar methodology, showed a progressive reduction in both basal and CoMTb-stimulated promoter activity with truncation of the construct from 1,612 to 642 bp in length (Figure 5C) compared to WT. The 1,612 bp truncation is missing a stromelysin platelet-derived growth factor responsive element (SPRE; −1,659 to −1,643 bp) and part of the stromelysin IL-1 responsive element (−1,614 to −1,595 bp), and in addition, the 642 bp truncation is missing 4 AP-1, 2 STAT3, and 1 c-rel binding sites.

### CoMTb-Driven MMP-1 Expression and Increased Histone Acetylation in the Promoter Region

**Figure 6:**
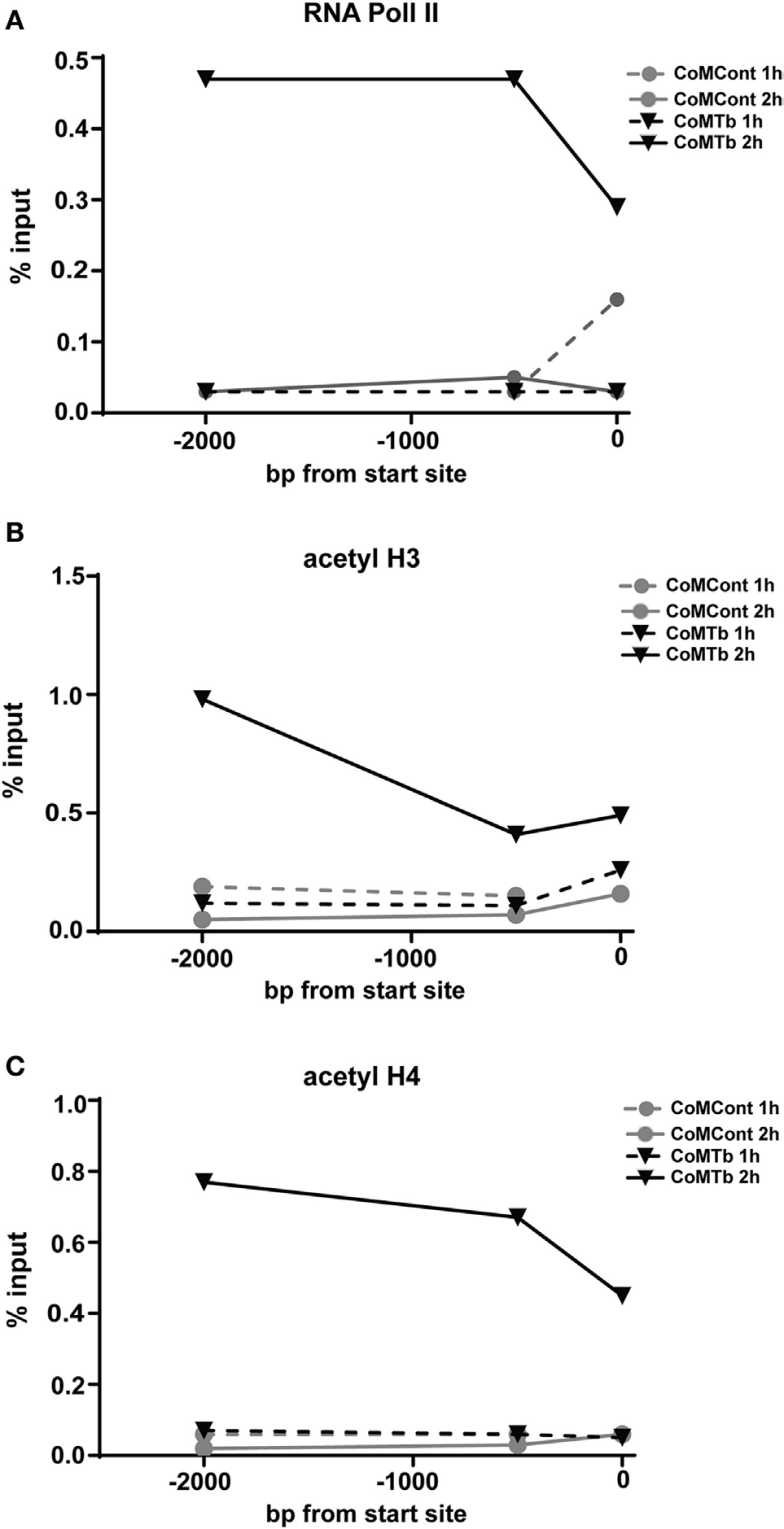
***Mycobacterium tuberculosis* (Mtb)-driven matrix metalloproteinase (MMP)-1 expression is associated with increased RNA Pol II binding and increased histone H3 and H4 acetylation of the MMP-1 promoter**. ChIP assays were performed on normal human bronchial epithelial cells treated with CoMTb (black triangles) or control medium (gray circles) for 1 h (dashed line) or 2 h (solid line) poststimulation. **(A)** RNA Pol II, **(B)** acetylated histone H3, and **(C)** acetylated histone H4 association with the MMP-1 promoter increases after 2 h CoMTb stimulation, particularly between −2,000 to −1,000 bp from the transcriptional start site. Data presented as percentage of the total chromatin input (% input) and figures are representative of three independent experiments. Acetyl H3, acetylated histone H3; Acetyl H4, acetylated histone H4; ChIP, chromatin immunoprecipitation; RNA Pol II, RNA polymerase II.

To further investigate whether epigenetic modifications of the MMP-1 promoter controlled the response to CoMTb, we examined the histone acetylation status of the MMP-1 promoter region by chromatin immunoprecipitation. Preliminary experiments using the respiratory epithelial A549 cell line suggested marked increases in histone H4 acetylation with CoMTb treatment at 1 and 2 h poststimulus (data not shown). In primary NHBEs, RNA Polymerase II binding to the MMP-1 promoter was increased between 10- and 15-fold in cells after 2 h of CoMTb treatment (Figure 6A). Histone H3 acetylation was increased 2 h poststimulation and was approximately threefold greater than under control conditions in the proximal promoter, and fivefold higher than control when measured 2 kbp upstream of the MMP-1 transcriptional start site (Figure 6B). Detection of acetylated histone H4 after 2 h of CoMTb stimulation was also increased across the MMP-1 promoter region. Histone H4 acetylation was 10-fold higher at −2 kbp and −500 bp, and 6-fold higher than unstimulated controls at the transcriptional start site (Figure 6C).

## Discussion

Upregulation of MMP-1 in TB is a critical event for the development of cavitation (9). Previous studies from our group and other have demonstrated that Mtb-stimulation upregulates MMP-1 expression, which is enzymatically active and able to degrade type I collagen, the main component of the lung’s ECM [Al (10, 28, 29)]. Expression of other MMPs is also induced in pulmonary TB (28, 30), and MMPs are implicated in central nervous system immunopathology (31, 32). In this study, we demonstrated that expression of MMP-1 and -3 in response to Mtb is controlled by epigenetic changes in histone acetylation. HDACs are canonically considered to be negative regulators of gene expression, and we showed a change in the profile of HDAC expression following Mtb infection of macrophages. Downregulation of Class I HDAC gene expression was observed with a concomitant and selective upregulation of HDAC4 but not HDAC5. In contrast, in respiratory epithelial cells, Class I and Class II HDAC expression were unchanged after CoMTb stimulation, implying that this is a cell type-specific effect. TLR signaling is known to induce changes in HDAC expression and activity (33), and our findings are consistent with published evidence of selective changes in macrophage HDAC expression in response to specific inflammatory stimuli (34). Our data show that Mtb infection of macrophages induces a change in the cellular HDAC profile. We went on to investigate the likely effects of this change by performing experiments in which MMP responses were examined under conditions of both general and specific HDAC blockade.

Non-selective HDAC inhibition using TSA and the bipolar hybrid CBHA suppressed Mtb-driven MMP-1 and -3 secretion and mRNA accumulation in primary epithelial cells and macrophages. Such a paradoxical effect on MMP expression has been previously reported in an *in vitro* model of arthritis, where chondrocyte collagenase activity was inhibited by TSA (35). In a mouse model of arthritis, TSA also inhibited MMP-1, -3, and -13 expression (36). Similarly, LPS-induced MMP expression by murine bone marrow-derived macrophages was inhibited by TSA (37). The Class I selective HDACi MS-275 had contrasting effects: increasing basal and CoMTb-stimulated MMP-1 and -3 secretion in epithelial cells, while in Mtb-infected macrophages, it decreased MMP-1/-3 secretion. Similar inhibition of cytokine-induced MMP-1 was seen in MS-275 treated human chondrocytes (38). The effects of MS-275 may be due to selective inhibition of HDAC1 at lower concentrations, and additional effects on the activity of HDAC2 and 3 at higher concentrations (39). Individual HDAC enzymes are likely to play different roles in regulation of MMP expression, and since these chemicals affect function of multiple HDACs, it is impossible to dissect the relative contribution of each HDAC by a chemical inhibition approach alone.

The increased secretion of MMP-1 and -3 from NHBEs observed with Class I HDAC inhibition, as compared to non-selective HDAC inhibition, is consistent with the premise that Class I HDACs are key negative regulators of MMP expression. HDAC1 was shown to be recruited to the MMP-9 promoter site, reducing histone H3 acetylation and NF-κB recruitment, leading to repression of MMP-9 expression in fibrosarcoma cells (40).

The enhancement of CoMTb-stimulated MMP expression observed with MS-275 led us to hypothesize that silencing the expression of HDAC1 might similarly increase inducible MMP expression. The catalytic activity of HDAC 1 and 2 is reliant on their incorporation as heterodimers into multiprotein assemblies (41) and therefore we expected that silencing either HDAC 1 or HDAC 2 would affect MMP expression. However, in spite of a high efficiency of HDAC1 silencing, MMP-1 expression was unaffected, and MMP-3 secretion was decreased in HDAC1-silenced cells. In contrast, upregulation of MMP-1 in CoMTb-stimulated conditions was further enhanced with HDAC2 inhibition, suggesting that MS-275 could be having its effect via inhibition of HDAC2 rather than HDAC1. The differing results for HDAC1 and HDAC2 silencing are unexpected given the close homology between these proteins, and consequently further dissection of their relative contributions to control of MMP expression is needed. In other experimental systems, different HDACs have been implicated in MMP regulation and there may be cell and stimulus specificity in host responses. In synovial fibroblasts from arthritis patients, HDAC1 siRNA enhanced TNFα-induced MMP-1 expression (42), whereas HDAC4 was identified as a negative regulator of MMP-1 expression (43).

Consistent with the hypothesis that epigenetic modifications regulate MMP secretion in TB, expression of MMP-1 and -3 were both suppressed by HAT inhibition with HATi II. Experiments using a structurally unrelated HAT inhibitor, AA, generated consistent data. Similarly, in human dermal fibroblasts exposed to UV light, AA inhibited MMP-1 expression, as did siRNA silencing of p300 expression. Increased HAT activity and histone H3 acetylation and decreased HDAC activity preceded changes in MMP-1 gene expression (44). The finding that both HAT and HDAC inhibition decreased MMP-1 and -3 expression, while apparently contradictory, may reflect the complex interdependence of these processes. It is well recognized that many non-histone substrates of these enzymes exist (45, 46) and indeed phylogenetic studies have indicated that bacterial HDAC homologs pre-date the existence of histones (47). Lysine acetylation of many non-histone proteins has been shown to be enhanced by HDAC inhibition, for example, with MS-275 (46).

Many transcription factors contain such lysine acetylation sites, including cAMP response element-binding protein (CREB), whose activity is increased in the presence of TSA (48). CREB can be acetylated at three sites, enhancing its transcription factor activity, and HDAC8 is known to act on the CREB acetylation sites (49). The NF-κB family of transcription factors is also subject to posttranslational modification including acetylation as well as phosphorylation (50). Myocyte enhancer factor-2 is deacetylated by HDAC3, which also acts on the HATs PCAF and p300/CBP (51). In addition to transcription factors and HATs, the HDACs themselves contain lysine acetylation sites, as do a number of structural and regulatory proteins (52). Therefore, it is difficult to dissect out the relative contribution of inhibition of histone acetylation/deacetylation compared to effects on these other substrates when considering the effects of chemical inhibitors, and our findings indicate a complex interplay of signaling pathways occurs during infection.

The promoter-reporter analysis showed that inhibitory elements located 4,372–2,942 bp upstream from the MMP-1 transcriptional start site decrease promoter activation, since deletion of this region enhanced CoMTb-driven promoter activity. The area between −2,942 and −2,001 bp contains several critical elements required for the induction of gene expression, including a putative NF-κB binding site. There is also an AP-1 site at −1,950 bp just proximal to the −2,001 bp truncation that might be functionally disrupted by this truncation. There is substantial evidence that MMP-1 expression is regulated by both NF-κB and AP-1 family transcription factors (8, 53, 54), and our data support a central role. Similarly, multiple transcription binding sites may be important in MMP-3 promoter function. Our chromatin immunoprecipitation studies demonstrated that CoMTb stimulation leads to increased H3 and H4 acetylation at the MMP-1 promoter region. Histone acetylation was an early event after CoMTb stimulation occurring concurrently with binding of RNA Pol II to the MMP-1 promoter.

In summary, MMP-1 and 3 expression in TB is regulated by HDAC and HAT activity. MMP-1 upregulation, as a result of epigenetic control, has the potential to drive tissue damage in the lung, thereby facilitating spread of infection and development of pathology. Chemical inhibition suggests that HDAC and HAT activity is necessary for inducible expression of MMP-1 and -3 in Mtb-infected macrophages, but that different mechanisms operate in NHBEs, where class I HDACs appear to act as a brake on collagenase expression. This is a selective effect, as MMP-7, which is constitutively expressed in MDMs and upregulated by Mtb infection, was unaffected by HDAC and HAT inhibition.

The minimal inhibition of MMP responses seen with siRNA targeting individual class I HDACs implies that there may be some redundancy of function. Increased histone acetylation in the MMP-1 promoter region follows Mtb stimulation, favoring RNA Pol II binding, and results in upregulation of MMP gene transcription and enzyme secretion. Tissue breakdown mediated by MMP activity is a key event in TB immunopathology and manipulation of host epigenetic changes have potential applications as host-directed therapy in the era of rising drug resistance in TB.

## Author Contributions

JF conceived the project. RM, PE, and JF designed the experiments and analyzed the data. RM, SB, and FS performed the experiments and generated the data. SB, RM, PE, and JF wrote the manuscript, which was reviewed and final version approved by all authors.

## Conflict of Interest Statement

The authors declare that the research was conducted in the absence of any commercial or financial relationships that could be construed as a potential conflict of interest.
