# Supplementary material for: Epigenetic Regulation of Matrix Metalloproteinase-1 and -3 Expression in Mycobacterium tuberculosis Infection
Source: Front Immunol. 2017 May 24;8:602. doi: 10.3389/fimmu.2017.00602 (PMC5442172; doi:10.3389/fimmu.2017.00602)
Supplement: Supplementary file 1 [file presentation_1.pdf]

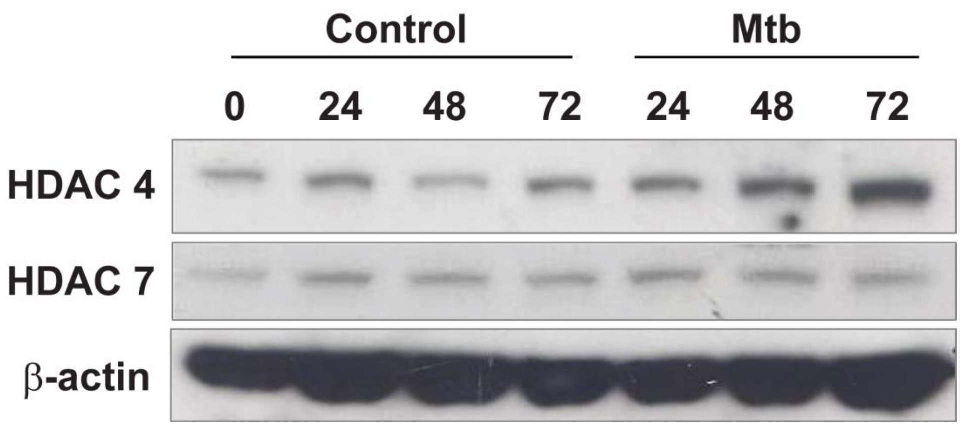

Figure S1- Macrophage HDAC4 protein expression increases in response to Mtb-infection.

Macrophages were infected with H37Rv at MOI 1 for 24 to 72 hours and Western blots were performed on the cell lysates. β-actin was used as a loading control. HDAC 4 protein expression increased in Mtb-infected cells at 48 and 72 hours post-infection, while HDAC7 was stably expressed.

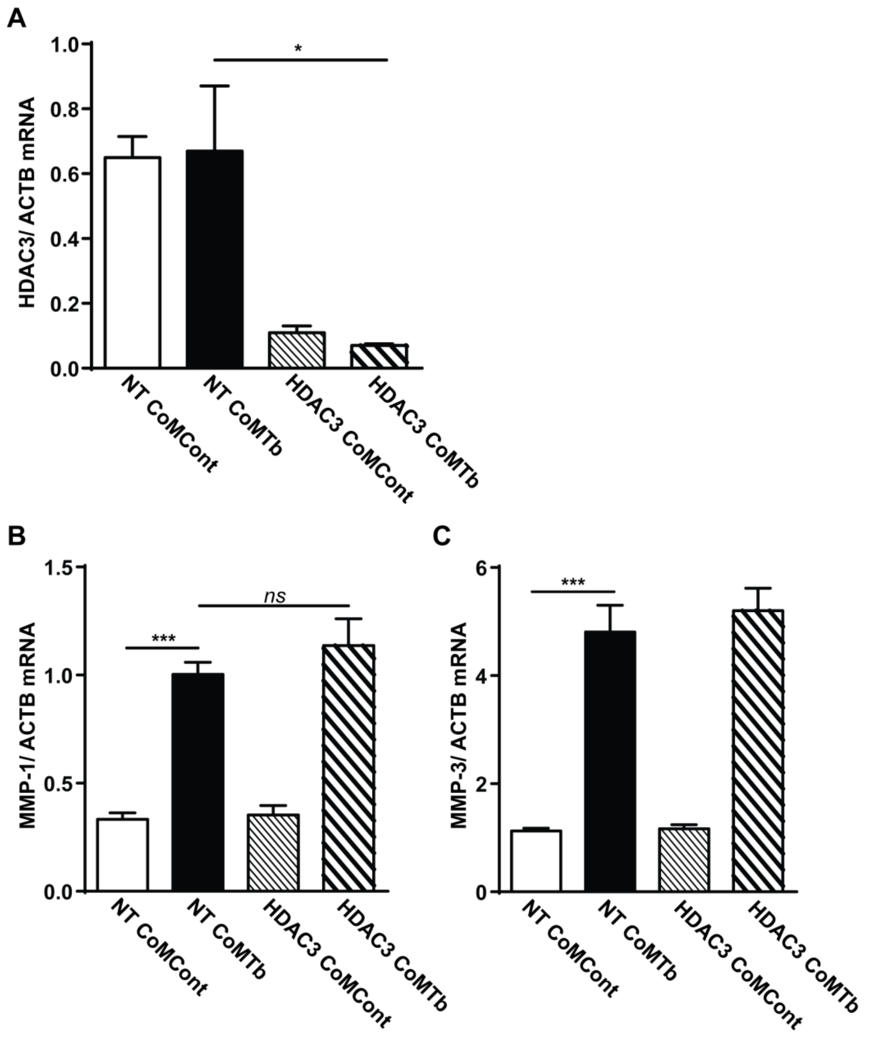

Figure S2- Silencing of HDAC3 expression does not inhibit CoMTb-driven MMP-1 and -3 gene expression.

NHBEs were transfected with 30nM non-targeting (NT) or HDAC3 specific siRNA. Cells were stimulated with CoMTb (1:5) for 24h. (A) HDAC3 mRNA normalized to the reference gene ACTB shows siRNA suppressed mRNA levels. (B) MMP-1 mRNA accumulation and (C) MMP-3 mRNA accumulation remained unchanged following HDAC3 silencing. mRNA of target genes was normalized to mRNA of the reference gene ACTB. Bars represent mean ± s.d. and analysis was performed using one-way ANOVA with Tukey's post-test.*$p<0.05$; ***$p<0.001$; ns- non significant. ACTB-beta-actin; HDAC3- histone deacetylase 3 siRNA; NT- non target siRNA.

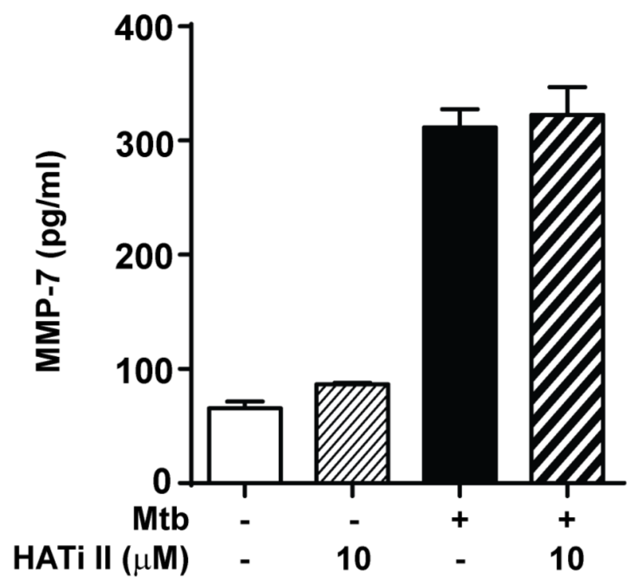

Figure S3- HATi II does not affect Mtb-driven MMP-7 secretion.

Macrophages were pre-incubated with 10µM HATi II prior to infection with H37Rv (MOI 1) and cell culture supernatants were collected after 72h. Pre-treatment with the HATi II did not affect MMP-7 secretion by Mtb-infected macrophages. Bars represent mean ± s.d. and analysis was performed using one-way ANOVA with Tukey's post-test.

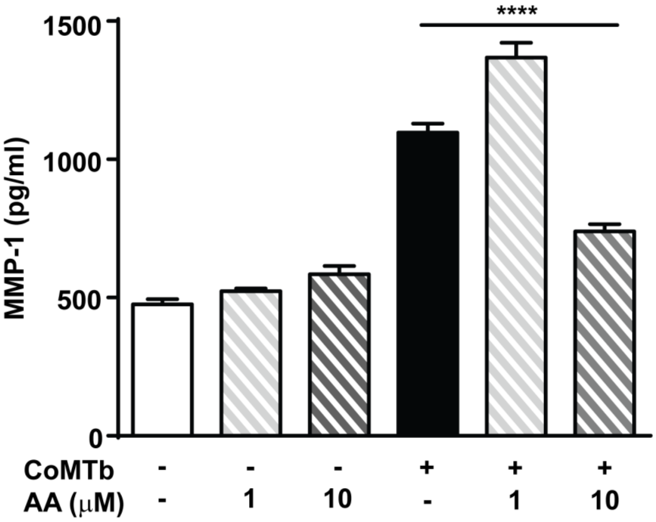

Figure S4- The HAT inhibitor Anacardic acid (AA) inhibits CoMTb-driven MMP-1 secretion in NHBEs.

NHBEs were pre-incubated with 1-10µM AA prior to CoMTb stimulation. Supernatants were collected after 72hrs and MMP-1 measured by ELISA. CoMTb-induced MMP-1 secretion is significantly inhibited by AA 10 µM. Bars represent mean ± s.d. and analysis was performed using one-way ANOVA with Tukey's post-test. ****p<0.0001; AA= anacardic acid.
